# Hydrogels and Carbon Nanotubes: Composite Electrode Materials for Long-Term Electrocardiography Monitoring

**DOI:** 10.3390/jfb15050113

**Published:** 2024-04-23

**Authors:** Leszek Kolodziej, Olga Iwasińska-Kowalska, Grzegorz Wróblewski, Tomasz Giżewski, Małgorzata Jakubowska, Agnieszka Lekawa-Raus

**Affiliations:** 1 Faculty of Mechatronics, Warsaw University of Technology, 02-525 Warsaw, Polandgrzegorz.wroblewski1@pw.edu.pl (G.W.); 2 Faculty of Electrical Engineering and Computer Science, Lublin University of Technology, 20-618 Lublin, Poland; 3 Faculty of Mechanical and Industrial Engineering, Warsaw University of Technology, 02-524 Warsaw, Poland; malgorzata.jakubowska@pw.edu.pl; 4 Centre for Advanced Materials and Technologies, Warsaw University of Technology, 02-822 Warsaw, Poland

**Keywords:** carbon nanotubes, hydrogel, ECG electrodes, electrocardiography, long-term monitoring, CNT composites

## Abstract

This paper presents methods for developing high-performance interface electrode materials designed to enhance signal collection efficacy during long-term (over 24 h) electrocardiography (ECG) monitoring. The electrode materials are fabricated by integrating commercial ECG liquid hydrogels with carbon nanotubes (CNTs), which are widely utilized in dry-electrode technologies and extensively discussed in the current scientific literature. The composite materials are either prepared by dispersing CNTs within the commercial liquid hydrogel matrix or by encasing the hydrogels in macroscopic CNT films. Both approaches ensure the optimal wetting of the epidermis via the hydrogels, while the CNTs reduce material impedance and stabilize the drying process. The resulting electrode materials maintain their softness, allowing for micro-conformal skin attachment, and are biocompatible. Empirical testing confirms that the ECG electrodes employing these hybrid hydrogels adhere to relevant standards for durations exceeding 24 h. These innovative hybrid solutions merge the benefits of both wet and dry ECG electrode technologies, potentially facilitating the extended monitoring of ECG signals and thus advancing the diagnosis and treatment of various cardiac conditions.

## 1. Introduction

Cardiovascular health care has become a prime issue for many developed countries. Stress and inadequate nutrition negatively affect health, causing many chronic heart disorders. The most widely used diagnostic procedure enabling the identification of cardiac abnormalities is electrocardiography (ECG). Typically, the signal is recorded during an examination lasting no more than a few minutes. However, the patient’s heart disturbances may vary throughout days or weeks. For this reason, physicians often require continuous monitoring of ECG signals lasting 24 h or longer. However, these measurements are currently technically challenging to perform. The standard procedure of a 12-lead ECG recording involves applying ten individual electrodes to the patient’s body at appropriate locations and then connecting a measurement cable from the ECG monitor to each point. The electrodes are composed of a hydrogel that collects the signal from the patient’s body, a contact usually made of silver (Ag)/silver chloride (AgCl) that transmits the signal to the test lead, and a skin adhesive that surrounds the hydrogel and holds the electrode in place. The hydrogel is the only conductive component of an electrode that contacts the skin. Therefore, it has a significant impact on accurate ECG recording. Most currently used hydrogels are liquid hydrogels, i.e., soft gel scaffolds filled with water and salt (usually NaCl or KCl) solutions [1,2]. Water and salt solutions form electrolytes, which enable the conduction of electrical signals from the patient’s skin. Additionally, water wets the skin, thus decreasing its impedance. The wet and soft characteristics of the liquid hydrogels enable them to adhere well to the skin at a microscopic level, further improving the signal collection. According to the American standard ANSI/AAMI EC12:2000 (R2020) for disposable ECG electrodes, the impedance of the electrode measured by the gel-to-gel method (10 Hz frequency) must be lower than 2 kΩ [3]. In the gel-to-gel method, pairs of electrodes adhere together so that their hydrogels attach. On average, the impedances of commercial electrodes at the beginning of measurements are in the range of 50–440 Ω [4]. These values are several times higher than the impedance of other system components (connectors, cables, or Ag/AgCl contacts). During long-lasting measurements, hydrogels dehydrate, especially when in contact with increased human body temperature. The drying of electrodes causes an impedance increase, which results in enhanced signal noise. Due to these issues, liquid hydrogels are often replaced by solid hydrogels (liquid hydrogels are characterized by a lower viscosity than solid hydrogels). They usually dry slower and cannot “flow out” of the electrode area. However, solid hydrogels do not fully adapt to the micro-surface of the epidermis, are more challenging to apply to electrodes during production, and do not sufficiently wet the epidermis. Another solution to drying-out electrodes is the formation of dry ECG electrodes free of hydrogel components. These concepts, which are not yet commercially available, are based on the highly developed micro/nano surface that adapts to the micro-geometry of the patient’s skin [5]. The proposed solutions include polymer composites, such as polydimethylsiloxane (PDMS)/titanium/gold [6], carbon nanotubes (CNT)/PDMS [7,8], and polypyrrole/CNT [9]; polysiloxane with metallic nanoparticle/nanowire composites (based on silver, platinum, or gold [5]); or textile electrodes (graphene-clad textiles [10] or an ECG-shirt [11]). Unfortunately, the dry-electrode solutions also are not flawless [7,12,13,14,15,16]. Complex production processes and expensive materials significantly increase their costs and make them highly difficult to implement in large-scale production. Many of the proposed solutions may not pass the biocompatibility tests. Their potential to conform to the skin surface during prolonged ECG recordings is uncertain. They also do not moisturize the epidermis, which does not ensure low skin–electrode contact resistance [17].

In the following paper, we address the challenges related to the signal collection by the interface ECG electrode in long-term ECG monitoring by merging the advantages of both wet- and dry-interface electrode solutions and the hybridization of hydrogels with dry-electrode materials. The target would be to obtain composite electrode materials that are highly electrically conductive, keep the conductivity over a prolonged period, can ensure conformal attachment to the skin, moisturize the skin, and are simple to manufacture, inexpensive, and meet international ECG standards.

To reach the above goals, we tested a set of commercial liquid and solid hydrogels composited with CNTs. CNTs are one of the most commonly used dry-electrode materials due to their unique electrical properties, strength, and low cost [18,19,20]. Published studies confirm their good biocompatibility upon contact with the outer skin layer [21,22,23]. CNTs can be used as a powder and as macro-assemblies like fibers, films, mats, or arrays, where CNTs form macroscopic structures connected by van der Waals and friction forces [24,25,26,27]. To the best of our knowledge, only one publication has focused on manufacturing an ECG hydrogel with CNTs [28]. The proposed solution used only a solid hydrogel, which does not moisturize the skin well. It was also not shown whether the hydrogel produced in the laboratory and the overall hydrogel–CNT hybrid meet the international standards set for ECG electrodes. The lack of conformal attachment to the skin required the deposition of the CNTs on the stretched hydrogel. The CNTs used were single-walled CNTs, which are currently very expensive. The CNTs were deposited on the surface of the hydrogel in the form of a thin, misaligned CNT film. Such electrode arrangement requires a change in the design of the overall electrode patch. 

Taking into account the requirements of real-life ECG electrodes, in the following work, we focused on various commercial hydrogels, which are well-established, inexpensive, approved medical products. In the case of CNTs, we chose inexpensive multi-walled CNTs (MWCNTs) in the form of powders and aligned CNT films. MWCNT powders are most-often used to produce nanocomposites due to the high availability, low cost, and intuitive manufacturing approach that involves mixing [29,30]. As an alternative, we tested the possibility of using macroscopic, aligned CNT films, which may be produced inexpensively on a large scale. Until now, CNT films were tested in many polymer nanocomposites [26,31,32]. Their pre-existing, dense, aligned CNT network enables the formation of composites with a much higher loading fraction of CNTs than CNT powders. Therefore, these composites can be characterized by better mechanical and electrical properties than powder-based nanocomposites. Inspired by this approach, we tested the possibility of forming hybrid hydrogel structures by wrapping commercial hydrogels in a CNT film.

## 2. Results and Discussion

We approached the formation of a hybrid CNT ECG hydrogel by selecting an appropriate commercial hydrogel to be combined with CNTs.

### 2.1. Hydrogels Selection

The impedance of five samples of each hydrogel product were tested according to the ANSI/AAMI EC12:2000 (R2020) standard [3] (see Section 3.4 in Materials and Methods for more details). The results are presented in Table 1.

As shown in Table 1, hydrogels can have widely varying impedances. The results of the average impedance measurement for hydrogels from various manufacturers range widely from 200 to 2530 Ω. The variability within the groups for the 5-element sample ranged from 40 to 1000 Ω. Interestingly, the impedance results of some of the tested products were barely within the standard impedance limit for disposable ECG electrodes. Out of the liquid hydrogels, SignaGel had the lowest impedance of all of the analyzed products. Therefore, it was selected as the primary product for the hybridization experiments. Similarly, the KM30B solid hydrogel was chosen for the film-wrapping experiments due to its lowest impedance.

### 2.2. Hybrid Hydrogels Produced by Mixing with CNTs

The good repeatability and stability of the parameters of the prepared electrodes require that a homogeneous hybrid gel with an appropriate proportion of CNTs is obtained. Due to the specificity of the described CNT-production process, the final product is obtained in the form of macroscopic CNT powder agglomerates. However, the best electrical results are achieved with forms consisting of individual CNTs. Therefore, before hybridizing with hydrogels, it is necessary to adjust the parameters of the CNT-deagglomeration process appropriately. The most common CNT-deagglomeration technique is the ultrasound homogenization of CNT suspensions in organic solvents. However, this procedure itself may have a substantial influence on the performance of the final hybrid hydrogels. Firstly, if the homogenization time is too short or the amplitude is too low, the CNT agglomerates may not be appropriately split. At the same time, a process that is too long or too intense can lead to the breaking of the CNTs themselves. In both cases, the result is the very high resistance of the homogenized CNTs. The second issue to mention is the solvent choice, as the solvent residues may cause skin irritation, preventing the hybrid hydrogels from passing biocompatibility tests.

**Figure 1:**
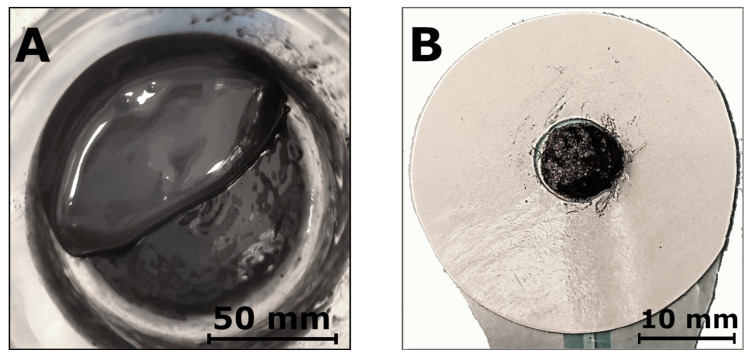
(**A**) Hybrid CNT hydrogel: SignaGel + 1% CNT (acetone) and (**B**) Hybrid CNT hydrogel: SignaGel + 1% CNT (acetone) on the electrode.

First, homogenization studies were performed using CNT array suspensions in a common organic solvent, acetone. The suspensions were homogenized for 15 min, 30 min, 45 min, 60 min, 90 min, and 120 min at 40% (approx. 50 W), 60% (approx. 74 W), and 80% (approx. 99 W) of amplitude and power. Increasing the amplitude to 80% resulted in a significant increase in temperature and the overheating of the probe during operation despite the process being conducted in ice. The powders produced using 40% and 60% were used to manufacture screen-printing pastes. The pastes were based on 8% PMMA in D.B. Acetate (see also Materials and Methods) according to a previously developed procedure. Five paths of 15 mm length, 1 mm width, and approx. 10 µm thickness (one layer) were printed using each paste. The paths were thermally dried, and their resistance was measured. All pastes made from powders homogenized at 40% power were characterized by a much higher resistance than the 60% ones. The average resistance measured for 60% power amounted to 46 ± 17 kΩ for 15 min and 52 ± 17 kΩ for 30 min. For 40% power, resistance was 2650 ± 350 kΩ for 15 min and 629 ± 172 kΩ for 30 min (see also Table S1 in Supplementary Materials). The lowest resistance was obtained for CNTs homogenized at a 60% power amplitude for 15 min. These nanotubes were used to produce the first hybrid hydrogels. The hybrid hydrogels were prepared by mixing CNT powders with SignaGel in a planetary mixer. The content of CNTs amounted to 0.1%, 0.5%, 1%, 2%, and 3% by weight. Using more CNTs affected the properties of the hydrogel structure, causing it to lose some of its features, such as its adherence to the skin and adaptation to its microstructure. Next, 185 ± 15 mg of the hybrid hydrogel was deposited on the previously printed silver-chloride electrodes. As previously mentioned, all tested electrodes were 10 mm in diameter. The hybrid CNT hydrogel and a sample on the electrode are shown In Figure 1.

**Figure 2:**
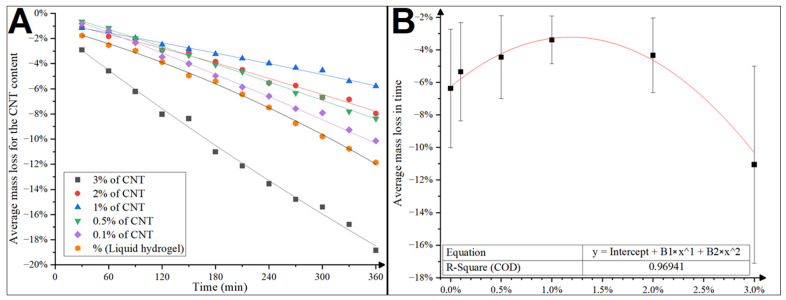
Comparison of the average mass loss of hybrid CNT hydrogels produced by homogenizing CNTs in acetone with polynomial regression (**A**). Average mass loss of hybrid CNT hydrogels in comparison to the SignaGel (**B**). Average mass decrease over time as a function of CNT content.

The drying of the hydrogels results in a loss of biosignal conductivity through the loss of skin contact caused by a decrease in product volume. Therefore, the slower the hydrogel evaporates water and thus loses its volume and mass over time, the longer it can work effectively in the ECG electrode. To determine the effect of CNTs’ presence on the drying of hydrogels, the hybrid hydrogels were subjected to mass loss tests. We conducted the tests using different batches of the same SignaGel hydrogel product. However, due to the large scatter of results between different batches of pure SignaGel, the comparative results presented were performed on one reference batch of the commercial hydrogel. Six electrodes of each produced series were adhered gel-to-gel, weighed, and placed on a heated table, which simulated the human body temperature. The samples were weighed every 30 min for 6 h. The humidity was kept constant at a 50% ± 5% level during the experiment. The results in the form of average percentage mass loss over time for each sample series are shown in Figure 2A,B (see also Figure S2 in Supplementary Materials for the other results).

**Figure 3:**
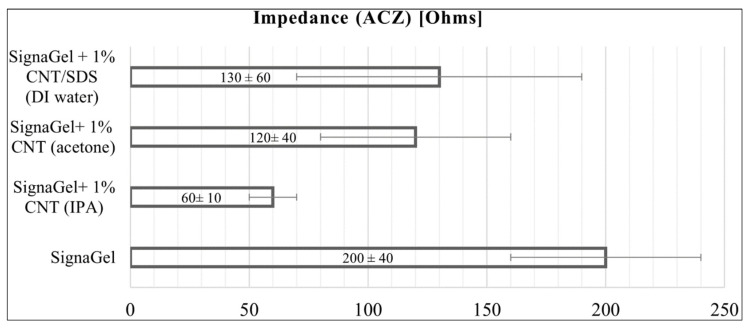
Results of impedance test of hydrogels produced by mixing performed according to the ANSI/AAMI EC12:2000 (R2020) standard.

The influence of CNT presence on the mass loss of the hydrogel was analyzed for five weight percentages (0.1%, 0.5%, 1%, 2%, and 3%) (see Figure 2B). A mass loss from 4% to 19% in 6 h was observed. The relationship was nonlinear, and the smallest mass loss and variability were observed for 1% of added CNTs. Therefore, the following four pairs of electrodes with this hybrid CNT hydrogel were produced. Samples of hybrid hydrogel with 1% CNT addition were subjected to electrical tests, according to the ANSI/AAMI EC12:2000 (R2020) standard, along with reference hydrogel samples. The Kruskal–Wallis test found that a statistically significant difference between all results was observed only in impedance measurements. It has been found that the average impedance of the electrodes decreased upon the addition of CNTs (Figure 3), and the difference is statistically significant (see also Supplementary Materials, Table S2, for full electrical test results).

The measurements showed a noticeable 40% decrease in the average impedance value for the hybrid hydrogel samples with the addition of 1% CNT relative to the reference non-hybridized hydrogels. Significantly, as expected, a stabilizing effect on impedance was observed and other electrical parameters were preserved. However, due to the possible harmful effects of acetone residues on the skin, skin-safe solvents were used in subsequent tests. This included isopropanol (IPA), the most common solvent in skin disinfectants, and a neutral-to-skin solvent-water. The manufacturing process of CNT hybrids using IPA was analogous to the acetone-aided process. The obtained samples were subjected to drying tests. For samples with 1 wt.% of CNTs, an average weight loss of −5.9% ± 2.5% after 6 h was obtained, which is almost the same as the results obtained for homogenization in acetone (a difference of 1.7%), and no significant statistical difference was found. Samples homogenized in IPA with the addition of 1% CNT were subjected to electrical tests, and the results are shown in Figure 3. The average impedance decrease, in this case, was as much as 70% compared to reference clear hydrogel.

Due to the strong hydrophobicity of CNTs, the sonication of CNTs in water required using a surfactant [33]. A common CNT surfactant SDS was selected for testing. Samples were made the same way as for acetone and IPA; however, in the case of water as a solvent before sonication, 0.3 wt.% of SDS was added. For the samples homogenized in water (with SDS) containing 1 wt.% of CNTs, the average mass loss amounted to −5.3% ± 0.8% after 6 h, which was also not a statistically significant difference (8.4% difference) from the results obtained for homogenization in acetone. Again, no significant statistical difference concerning reference samples was found. Therefore, samples with 1 wt.% CNT were selected for electrical tests. In the case of water as a solvent, an average impedance decrease of 35%, compared to reference clear hydrogel, was obtained.

As mentioned above, the mass loss of pure SignaGel upon drying differs between batches. Therefore, to ensure our results are reliable, we tested four additional batches of pure SignaGel and prepared hybrid hydrogels using these batches and CNTs homogenized in IPA and water with SDS. The average mass loss for the pure SignaGel was between −4.2% ± 1.4% and 7.6% ± 4.6%, for the CNT hybrid hydrogel homogenized in IPA it was −4.5% ± 1.5%, and for the CNT/SDS hybrid hydrogel homogenized in DI water it was −5.6% ± 1.3% (see also Table S3 in the Supplementary Materials). There was no statistically significant difference between any of the average results, which shows that, due to the large spread of results in the mass loss of the pure hydrogel, the addition of CNTs increases the stability of the hydrogel without impairing or enhancing its drying parameters. Stability is defined by the dispersion of the mass loss values for samples with 1% CNT added under the same conditions from the same batch. The initial mass loss of pure hydrogel depends on the batch of samples used for the test. After hybridization with 1% of CNTs, a mass loss of hybrid CNT hydrogel was always at the lowest possible level, not depending on the initial parameters of the hydrogel.

**Figure 4:**
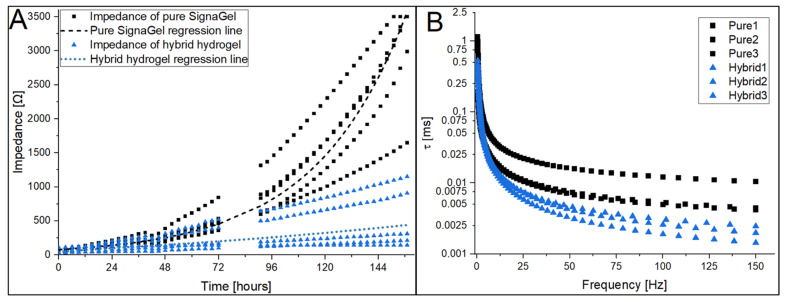
Results of (**A**) long-term impedance test of hydrogels and (**B**) time-constant parameter in frequency.

The hybrid hydrogel sample achieving the lowest impedance value, SignaGel + 1% CNT (IPA), was subjected to long-term impedance measurements. As in the other electrical tests, test leads were connected to a pair of electrodes, adhered by the gel-to-gel method, with either a hybrid or pure hydrogel (for reference). Then, the impedance was measured according to the ANSI/AAMI EC12:2000 (R2020) standard every three hours for 157 h. Measurements were made for five pairs of pure and hybrid samples. The results are shown in Figure 4A.

The measuring device required a technological break after 72 h of recording, hence the gap in data shown in the chart, which does not affect the other results. The difference between the results obtained in the long-term measurements is very significant. The median impedance of the pure hydrogel after 148 h is 2881.4 Ω, and for the hybrid hydrogel, 294.5 Ω. After this time, some pure hydrogel samples reached the maximum impedance indicated by the measuring device of 3500 Ω, although their actual impedance was higher. After 142 h, four out of five pure hydrogel samples exceeded the 2000 Ω impedance indicated by the standard as the limiting impedance for ECG electrodes, while all hybrid samples met the requirement throughout the test. The test took place under controlled conditions, and the electrodes remained static; however, in real-life situations on the patient’s body, the hydrogels may dry out much faster, and the advantage of using hybrid hydrogels is even greater. The graphs also indicate the exponential regression line fitted with a correlation coefficient of 0.97 and 0.68 for pure and hybrid hydrogels, respectively.

ECG electrodes record a signal with a specific frequency range that spans from almost DC signals up to 150 Hz [34,35]. The mentioned ANSI/AAMI EC12:2000 (R2020) standard focuses primarily on impedance measurements at the 10 Hz level. To test whether the hybrid hydrogels exhibit enhanced performance over a wider frequency range, we measured the impedance over the 0.5–150 Hz range, the most important part of the ECG signal [36,37]. This ensures a reliable measurement for the impedance analyzer used in this study. In the study, three samples, each of pure SignaGel hydrogel and hybrid SignaGel + 1% CNT (IPA), were adhered gel-to-gel and measured on an IM3590 analyzer. All samples show a clear exponential decrease in the impedance modulus with increasing frequency, and a statistical difference is observed between the CNT-enriched samples and pure hydrogel in the whole range of frequencies (see Table S4 in Supplementary Materials). Taking into account the fact that the total impedance of the ECG hydrogel material is represented by a series model of resistance and capacitance, we may expect that the capacitance will introduce time delays and thus a distortion of the detected signal. For a series connection of resistance and capacitance, we may write the following general equation:

(1) $${V=v}_{c}(t)+\mathrm{RC}\frac{{\mathrm{dv}}_{c}\left(t\right)}{\mathrm{dt}}$$

where V is the detected signal, $v_{c}$ is the voltage increase in the capacitance, R—resistance of the circuit and C—capacitance. By solving this equation, we obtain the following:

(2) $$v_{c}{=V(1\;-\;e}^{-\frac{t}{\mathrm{RC}}})$$

where RC represents a time constant (τ), after which the voltage on the capacitor reaches about 63% of its target value.

The longer the τ, the longer the transient state lasts in the circuit, so the lowest possible value of the time constant is desirable in ECG electrodes. The determined τ in the frequency range, on a logarithmic scale, is presented in the graphs in Figure 4B.

A statistical analysis using the Kruskal–Wallis test showed a significant statistical difference between the measured data series. On average, the τ value for pure hydrogel samples is 0.1% to 326% higher than for hybrid samples, increasing with the frequency. Thus, the results show better properties of the hybrid samples over a wide range of frequencies, indicating lower distortions of the recorded ECG waveforms.

**Figure 5:**
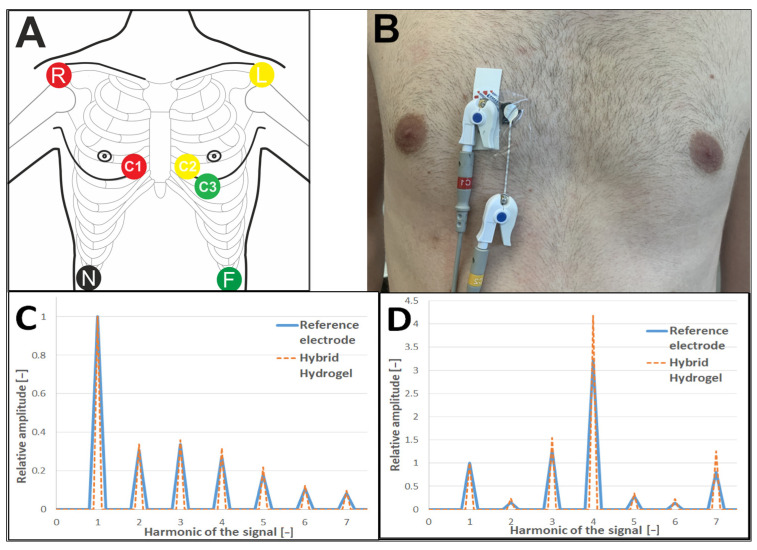
(**A**) Scheme of electrodes placement; (**B**) photo of one pair of hybrid hydrogels and reference electrodes; FFT analysis of recorded waveform of the C2 ECG signal, before (**C**) and after 72 h of recording (**D**), in absolute units.

The hybrid hydrogel SignaGel + 1% CNT (IPA) was also tested in vitro. The test was performed on one patient using the seven-lead electrode system. Four standard wet electrodes (FES-5541C) were applied to the body of a healthy patient on the limbs in the Mason-Likar arrangement [38]. The same electrodes were also used as a reference at the C1-C3 position [39,40]. Then, as in previous tests, a small amount of hybrid hydrogel (185 + 15 mg) was applied to the Ag/AgCl electrodes, and the electrode was applied to the body at a short distance (about 5 mm) from the reference electrode. Chest electrodes remained on the body for 72 h, and limb electrodes were replaced with new ones at the end of the measurement for more reliable results. A diagram of the electrode placement and a photo of one pair of electrodes placed can be seen in Figure 5A,B.

Two tests were performed on the electrodes. First, the ECG signal of the C1 hybrid and reference electrodes was measured immediately after the electrodes were applied, and fundamental analysis was performed. Fast Fourier Transform (FFT) analysis was conducted in the second test to transform signals from the time domain to the frequency domain.

The recorded waveform of the ECG of C1 electrodes (see Figure S3 in Supplementary Materials) is almost identical to the reference electrode signal. The average Pearson correlation coefficient was 0.985. The slight difference in amplitude is mainly due to the shifted position of the electrodes on the body. In order to avoid hiding the differences between the signals, only a basic filtering of 50 Hz power interference and 25 Hz muscle interference was applied, resulting in the apparent drift of the isoline. In addition, the determined SNR was 53.1 dB for the reference electrodes and 54.4 dB for the hybrid hydrogel.

We also subjected the recorded signals to FFT analysis. By obtaining signal data in the frequency domain, we were able to determine the first harmonic corresponding to the heart frequency, which was 2.0 ± 0.2 Hz for both measurements. The amplitudes of the all signal peaks are divided by the first harmonic. The data obtained by this analysis are easy to compare by the signal peaks for individual harmonics. An example of FFT analyses of the same electrode at baseline and 72 h after the start of measurement is shown in Figure 5C,D. The full results of the FFT analysis are provided in Table S5 in Supplementary Materials. As can be seen in the charts and data, in most cases, the amplitude of the signals recorded with the hybrid electrodes is higher than that of the referenced electrodes.

However, the results from the analysis of the ECG signals recorded in a living body are only partially indicative. The measured signal under natural conditions is affected by many factors, such as skin and ambient humidity, the place where the electrode is applied, the local absorption of water through the skin, the secretion of sweat, or the bending of the skin during the measurement. These uncontrollable factors, sometimes varying even for electrodes close to each other on the same body, affect the final results. Thus, the much better results obtained in previously presented lab tests, and no worse results in in vitro tests, prove that hybrid electrodes perform better than traditional electrodes.

The presented data show that hybridizing commercial hydrogels with CNT powders may be a simple and inexpensive way of improving the operation of ECG electrodes. The hybrid hydrogel remains liquid and can be deposited on the electrodes like a pure liquid hydrogel. The presence of the hydrogel enables the moisturization of the epidermis and good adhesion to the skin, resulting in an effective signal collection. The presence of CNTs decreases the impedance of the gel at a wide range of frequencies and signal distortions due to capacitive properties. The impedance improvement takes place for any solvent used to deagglomerate CNTs, including the solvents commonly used in medical applications. Finally, CNT addition improves hydrogel stability over time. Therefore, the impedance should be kept at a low level for a much longer time than in the case of reference samples.

### 2.3. Hybrid Hydrogels Manufactured by Wrapping Structures in CNT Film

**Figure 6:**
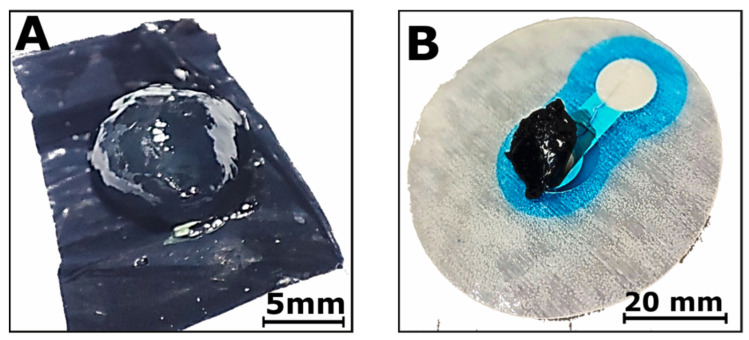
(**A**) Hydrogel placed on a piece of CNT film. (**B**) Wrapped hybrid CNT hydrogel on the electrode.

The second method of manufacturing hybrid CNT hydrogel structures involved using CNT films. CNT films have the property of clinging easily to various surfaces. Therefore, we decided to use it to create a pillow-like structure, with the CNT film acting as a cover for the soft interior made of liquid hydrogel. A portion (185 ± 15 mg) of SignaGel hydrogel was applied to a flat rectangle film structure, as shown in Figure 6A. Next, the hydrogel was wrapped in a film by folding the film corners. This structure retained a liquid hydrogel’s flexibility while evolving into a more compact product, which can be easily transferred into the electrode contact field (Figure 6B).

The film-wrapped structures were further tested to compare their properties to reference the wet liquid hydrogel. First, a pressure test was performed. When force is applied to a standard liquid hydrogel, it spreads over the surface on which it is placed. This can lead to a situation where the hydrogel flows away from the electrode area and no longer performs as an electrical conductor. A hybrid, CNT-wrapped hydrogel may introduce improvement in this respect. To check this property, a hybrid CNT film liquid hydrogel was pressed between two glass surfaces with a force of 20 ± 5 N.

**Figure 7:**
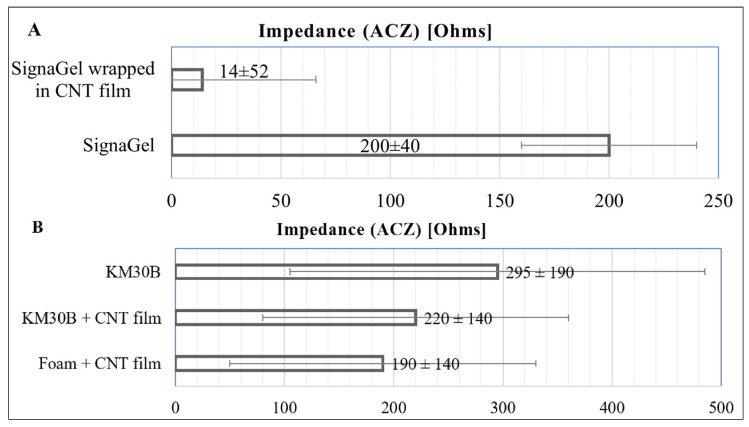
Results of impedance test of hydrogels performed according to the ANSI/AAMI EC12:2000 (R2020) standard, produced by (**A**) mixing or (**B**) wrapping solid structures.

The pressure tests showed that the structure retains its integrity until the film’s continuity is breached due to high-pressure application (above 50 N). However, the visible impermeability of the CNT film may cause concerns about the moisturization of the epidermis and sweat penetration. Therefore, drying tests were also performed. The hybrid hydrogels were placed on electrodes, and similarly to the previous experiments, they adhered gel-to-gel, and their mass loss was tested. The result of the mass loss test was −8.0% ± 2.6%. This result is within the margin of error for the pure hydrogel. Depending on the initial parameters of the pure hydrogel samples, hybridization can either improve or maintain hydrogel drying levels. This shows that hybridization by wrapping in CNT film, as in the case of hybrid hydrogels obtained by mixing, can elongate its drying period and increase its stability. Also, mass loss tests show that because the molecular water from the hybrid hydrogel exchanges with the surroundings, the skin will be locally moisturized during the measurement, as for a standard liquid hydrogel. This may be explained by the nanoporous carbon structure of CNT assemblies. which was found before to adsorb molecular water [41]. Further, electrical tests of the wrapped hydrogels were performed using the gel-to-gel method (see also Supplementary Materials Table S6). The difference in the ACZ test is presented in Figure 7A.

Despite the scatter of results, there was a significant statistical difference for most of the obtained values, not only in the impedance test. There was a significant improvement in the achieved results. The average impedance decreased even by 93%. Other electrical parameters also exhibited much lower values than for the product before hybridization.

To test the role of the hydrogel component in the CNT-wrapped hydrogel, comparative studies were performed by wrapping the disc (10 mm diameter) of solid hydrogel KM30B and medical adhesive foam in CNT film and testing the samples by the same procedure. The difference in the ACZ test is presented in Figure 7B (see also Supplementary Materials Tables S6 and S7 for complete electrical tests results). The results obtained for the solid structure packing were noticeably worse than for the hybrid liquid hydrogel CNT film. Additionally, although the wrapping of the solid hydrogel and the medical foam improved the electrical parameters of the standard solid hydrogel structure, the advantages of traditional wet hydrogels were lost.

CNT-wrapped solid hydrogels could not moisturize the epidermis and could not adhere well to the skin.

### 2.4. Model of Electrical Conduction of the Wrapped Hybrid CNT Hydrogel

As long as the conduction improvement of the hydrogels mixed with CNT powders may be explained by the percolation models used for other CNT nanocomposites, the conduction mechanism of the wrapped hybrid structures is only partially obvious. Therefore, a set of experiments was conducted to precisely understand the mechanism of the wrapped structures’ conduction.

Firstly, the resistivity of the CNT film, hydrogel, and hybrid CNT liquid hydrogel was determined. The samples were placed in a purpose-designed container, limiting the volume of the samples to 20 × 5 × 1.1 mm^3^. Due to the constant known height of the CNT film, it was limited to only two dimensions. At the ends of the container were silver contacts for measuring the resistance. In this test, CNT film was not wrapped around the hydrogel but stacked below and over the hydrogel. In addition to measuring the resistance of the hybrid, a theoretical value of resistance was calculated by assuming a parallel combination of resistors, i.e., the CNT film and the SignaGel layer. The determined resistivity is shown in Table 2.

The determined value of resistivity of the hybrid CNT stacked liquid hydrogel differs from the calculated one by less than 4 mΩm, which, after considering the measurement error, confirms the assumption of the parallel combination of resistances in the substance model. Using Ohm’s law relation, the current flow through the individual components of the hybrid CNT stacked hydrogel model was determined. Due to the significant difference in resistivity between SignaGel and CNT film, 99.95% of the electric charge flows through the CNT film. This means that hybrid CNT-wrapped hydrogel had a resistance nearly equal to pure CNT film. The slight difference in resistivity values between the calculation and experiment is mainly caused by the wrapping process.

Previous reports showed that the electrical properties of CNTs may be significantly affected by molecular water (humidity) and water in liquid form [41,42,43,44]. Electrolytes could also potentially influence the conductivity of the films. Therefore, further studies were performed. Since the wet hydrogel is in continuous contact with the CNT film, the impact of humidity on the resistance of the CNT film was investigated. For this purpose, the ends of the CNT film adhered to two silver contacts so that the middle part of the film floated in the air (the film samples were cut along the nanotube axial alignment). The structure prepared this way was placed in a container where the humidity was maintained at 95–100% for 72 h. The electrical resistance of the film samples was measured every 10 min. It was observed that the film resistance decreased for 25 ± 5 h, reaching a result 17% ± 3% lower than the initial value, and maintained this level until the end of the measurement. Therefore, it may be concluded that the exposure of the CNT film to the molecular water from the wet hydrogel has a positive effect on the electrical parameters of the hybrid CNT-wrapped hydrogel by lowering its electrical resistance.

**Figure 8:**
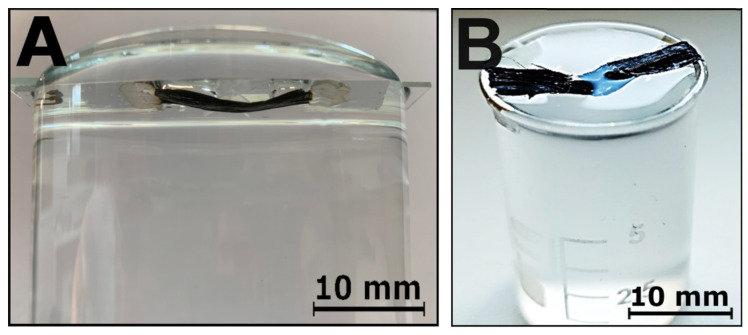
(**A**) CNT film on glass in DI/KCL mixture; (**B**) CNT film bridge in DI/KCL mixture.

Further, the effect of the direct exposure of the CNT film to individual hydrogel components, liquid water and a water–salt mixture, was studied. CNT film fragments were immersed in pure DI water and in DI water with NaCl and KCl salt mixtures. As in the previous tests, in the first part of this experiment, a ribbon of a CNT film sample was adhered by its ends to the laboratory slides using silver paste. Then, the samples were placed on the containers with DI water mixtures with NaCl and KCl of 10% concentration so that the most significant possible part of the film was immersed without immersing the measuring contacts, as presented in Figure 8A. A series of test specimens was also left in the air. In the second part of the experiment, the pieces of the film adhered directly to the glass vessel at its edges to form a bridge immersed in the liquid to eliminate possible interference caused by covering the vessel with a glass plate in the first part of the experiment, as presented in Figure 8B. A series of test samples was also placed in pure DI. The resistivity of all the films was measured for 24 h; the average results are shown in Table 3.

No significant decrease in resistivity for any analyzed sample series was observed. The differences between specific results are caused by the measurement error. The reduction in resistance between samples immersed in different salt mixtures was not significantly different from test samples, so neither the type of salt used nor the immersion method affects the decrease in resistivity.

## 3. Materials and Methods

### 3.1. Materials

**Hydrogels**: Four commercially available wet liquid hydrogels were used: Spectra 360 electrode gel manufactured by Parker Laboratories Inc., Fairfield, NJ, USA; ECG&EEG gel purchased from E.F. Medica Srl. Transound, Italy; SignaGel from Parker Laboratories Inc., USA; and ECG gel from Żelpol Ltd., Warszawa, Poland. Solid hydrogels included KM10B (0.81 ± 0.13 mm) manufactured by Katecho LLC., Des Moines, IA, USA; HIT-B3M (0.60 ± 0.15 mm) produced by Sekisui Kasei Co., Ltd. St-Gel, Osaka, Japan; and RG-63X Hydrogel from Cardinal Health Inc., Dublin, OH, USA. For some reference tests, medical single-coated foam MED 5635, manufactured by Avery Dennison Corporation, Ireland, was also used. MED 5635 foam has a similar structure to a solid hydrogel while being a non-conductive material.

**Solvents and surfactants**: The following solvents and surfactants were used for CNT sonication: acetone (≥99%) and 2-Propanol (IPA, ≥99%) obtained from Chempur, Poland and sodium dodecyl sulfate (SDS) ultrapure (≥99%) surfactant obtained from Carl Roth GmbH + Co. KG, Karlsruhe, Germany, dissolved in double distilled water (DI).

**CNT powders**: CNT powders were obtained in a two-stage process. First, CNT arrays (also referred to as carpets or forests), i.e., macroscopic CNT structures in which individual CNTs are arranged vertically and closely packed, were produced via the floating catalyst chemical-vapor-deposition (FCCVD) process described previously [24,26]. In brief, the CNT arrays were made in a horizontal reactor with a one-meter-long quartz tube of a 75 mm diameter and a three-phase heating system. In brief, ferrocene and toluene-containing feedstock were injected into the hot zone of the reactor (temperatures above 600 °C) under an argon flow. In these conditions, ferrocene and toluene pyrolyse release iron and carbon atoms, respectively. Iron atoms combine into clusters and deposit on the walls of the reactor. Carbon nanotubes are synthesized on the catalysts in the form of densely packed MWCNT arrays. In the second stage, CNT arrays were deagglomerated into CNT powder form via a homogenization process. The nanotubes and chemical compounds were weighed on a Radwag AS 220.X2 PLUS analytical balance. The CNT arrays were mixed with solvents at a ratio of 1:1000 by weight. The deagglomeration process was carried out in an ultrasonic homogenizer (Sonics VibraCell™ VCX 500) at a 20 kHz frequency with a 0.5″ probe. During homogenization, beakers with samples were cooled with ice due to the exothermic character of the process. Further, the solvents were evaporated using the Memmert UF55 oven, manufactured by Memmert GmbH + Co. KG, Schwabach, Germany.

**CNT films**: CNT films were obtained via another FC-CVD process described previously [25,26]. In this process, a feedstock containing methane, ferrocene, and thiophene is injected into the hot zone of a horizontal (or vertical) reactor. At temperatures above 1000 °C and a reduced hydrogen atmosphere, the CNTs grow and form an elastic cloud (aerogel). The cloud is then extracted at the bottom of the reactor using a metal rod. Due to the temperature gradient, the nanotubes attach to the rod, forming a partly aligned transparent CNT film, which is then wound on a spindle layer by layer. The final product is a CNT film of approx.—10 µm in thickness and tens of centimeters in length and width.

### 3.2. Preparation of CNT Screen-Printed Paths

To prepare screen-printing CNT pastes, homogenized CNT powders were mixed with 8 wt.% polymethyl methacrylate (PMMA, obtained from BASF GmbH, Heidelberg, Germany) in butyl diglycol acetate (D.B. acetate, CAS: 124-17-4, obtained from Sigma-Aldrich Ltd., St. Louis, MO, USA) [30]. They were ground in a mortar for 10 min and then unified in a three-roll mill Exakt 80E with 80 mm diameter SiC rollers manufactured by EXAKT Technologies, Inc., Oklahoma City, OK, USA [45]. The unification of all samples was performed with a 5 µm gap. After screen printing using a screen and stencil printing machine, C920, manufactured by AUREL SPA, Italy, printouts were thermally dried at 120 °C for 30 min in a Memmert UF55 oven.

### 3.3. Preparation of Hybrid Hydrogels Reference Electrodes

Reference electrodes were made of thermoplastic polyurethane (TPU) film substrates of 120 µm thickness produced by Elecrom Stretch Clear from Policrom Screens SPA, Italy. On the substrates, silver paths (100 mm length, 2 mm width) were screen printed with silver ink Loctite Edag 725A(6S54) produced by Henkel AG & Co. KGaA, Germany. Next, measurement contacts (10 mm diameter) were coated with silver chloride ink LOCTITE EDAG 6017SS E&C produced by Henkel AG & Co. KGaA Germany. After both screen-printing procedures, printouts were dried at 120 °C for 20 min. After that, medical silicone tape (50 mm in diameter) with a 10 mm diameter hole in the middle, 2477P, double-Sided silicone/Acrylate produced by 3M, USA, was applied around the measurement pads. Finally, a portion (185 ± 15 mg) of the liquid hydrogel or a disc (10 mm) of the solid hydrogel was deposited on each measurement pad. These samples were then adhered gel-to-gel and subjected to examination.

Hybrid hydrogels with CNT powders were obtained by weighing out the appropriate portions of hydrogel and CNT on the mentioned Radwag scale, initially mixing by hand, then 3 stages of 1 min each of mixing in a Planetary Mixer Kakuhunter SK-350TII manufactured by Shashin Kagaku Co., Ltd., Kyoto, Japan, at 500, 1000 and 2000 rpm, were performed, respectively. Finally, the unification process on the mentioned three-roll mill with a 5 µm gap was carried out for additional homogenization.

The CNT film for the wrapped hybrid hydrogel was cut with a scalpel into 20 ± 5 mm squares and manually wrapped around a portion of the hydrogel.

### 3.4. Characterization of Composite Materials and Electrodes

The electrical parameters of electrodes have been tested according to the ANSI/AAMI EC12:2000 (R2020) standard for disposable ECG electrodes by a SEAM CALM electrode tester from Q.C. Integrated Solutions. The standard specifies five electrical parameters: A.C. impedance (ACZ); D.C. Offset voltage (DCO); combined Offset Instability and internal noise (NOISE); defibrillation overload recovery (SDR); Bias current Tolerance (BIAS) internal noise, Offset voltage, defibration overload recovery, and impedance testing. The individual tested electrode pairs must provide results below the critical values indicated in the standard in the specified test sequence. For example, in the ACZ test, the average value of 10 Hz impedance for electrode pairs connected gel-to-gel at a level of impressed current not exceeding 100 µA peak-to-peak (pp) shall not exceed 2 kΩ. A detailed description of the other test can be found in the Supplementary Materials.

Additional resistance measurements were performed using Multimeter Keysight 34465A manufactured by Keysight Technologies, Inc., Santa Rosa, CA, USA.

Serial resistance and serial capacitance measurements to determine the time constant of the samples were made on a chemical impedance analyzer IM3590 manufactured by Hioki E.E. Corp., Dallas, TX, USA.

The ECG signal waveform was recorded with an M-Trace Pc ECG device manufactured by M4Medical Ltd., Lublin, Poland, using FES-5541C electrodes produced by FARUM Ltd., Warsaw, Poland. An ECG signal was recorded on one patient, and ethical approval for this study was obtained from the Ethics of Research with Human Subjects Team of Warsaw University of Technology, Warsaw, Poland.

For hydrogel-drying tests, the plate of a magnetic stirrer (RCT basic manufactured by IKA-Werke GmbH & Co. KG, Staufen im Breisgau, Germany) heated to 36.6 °C, simulating human body temperature, was used. During the test, samples were weighed on the mentioned Radwag scale.

### 3.5. The Statistical Analysis

The effect of the hybridization of hydrogels with CNTs on their electrical properties and stability performance was statistically analyzed. The factor analyzed was the mass percentage of CNTs in the hybrid CNT hydrogel. The statistical analysis was performed using variance checks, the Kruskal–Wallis test, and Mood’s median test in the STATGRAPHICS Centurion 19 software. Errors bars in graphs and tables correspond to the maximum and minimum values from an average for a presented data series. Five samples of relevant materials were tested for statistical sampling in each of the presented tests. The graphs and ECG signal analysis were performed with Origin 2022 software by OriginLab Corporation (Northampton, MA, USA), and the value of the signal-to-noise ratio (SNR) was calculated using Matlab software 2023a by The MathWorks, Inc. (Natick, MA, USA).

## 4. Conclusions

In this study, we have demonstrated the potential for enhancing the performance of commercial ECG hydrogels through hybridization with carbon nanotubes (CNTs). We established that incorporating a small amount (1%) of CNTs into traditional ECG gels markedly reduces their impedance across the spectrum of frequencies typical of ECG signals while simultaneously decreasing signal distortion by reducing the hydrogel’s capacitance and the circuit’s time constant. The hybrid hydrogel maintains the skin adherence and epidermal wetting characteristics of conventional hydrogels but benefits from the improved stabilization of its electrical properties upon drying, thereby extending its operational lifespan.

Furthermore, our findings indicate that the hybrid hydrogels can be applied onto electrodes in a manner similar to pure hydrogels, using skin-safe solvents to preserve biocompatibility. In vitro testing confirmed the effectiveness of these hybrid gels.

Additionally, our research suggests an alternative, possibly more promising method of hybridization, involving the use of CNT films. Encasing either liquid or solid hydrogels with CNT films substantially enhances their electrical parameters, as measured by the ANSI/AAMI EC12 standard for disposable ECG electrodes. This method results in a more compact hybrid that is easier to deposit on electrodes while maintaining the flexibility of the wrapped hydrogels. Our experiments demonstrated that, although the CNT film encases the hydrogel, it permits water exchange, thereby moisturizing the epidermis. Most significantly, conductivity analyses indicate that the majority of electrical conduction occurs through the CNT films, with the liquid hydrogel and its electrolytes enhancing the film’s conductivity.

Given these results, particularly with the CNT film-wrapped hydrogel hybrids, there is significant potential for the broader application of these developed electrodes. An intriguing possibility is their use in electroencephalography (EEG) monitoring, where the challenges of low impedance and electrode drying are more pronounced than in ECG applications.

## Figures and Tables

**Table 1:** Results of impedance tests of commercial liquid and solid hydrogels according to the ANSI/AAMI EC12:2000 (R2020) standard.

Liquid Hydrogels	Average Impedance (Ω)
SIGNAGEL	200 ± 40
SPECTRA 360	1890 ± 1000
TRANSOUND	2530 ± 900
ŻELPOL	570 ± 400
**Solid Hydrogels**	**Average Impedance (Ω)**
KM30B	230 ± 25
HIT-B3M	1100 ± 450
RG-63X	1000 ± 540

**Table 2:** Average resistivity of CNT film and hydrogels.

Substance	Thickness	Resistivity
SIGNAGEL	1.1 ± 0.1 mm	5.33 ± 4.02 Ωm
CNT Film	10 ± 1 µm	478 ± 157 µΩm
Calculated value hybrid CNT stacked liquid hydrogel	1.1 ± 0.1 mm	2.86 mΩm
Measured value hybrid CNT stacked liquid hydrogel	1.1 ± 0.1 mm	6.84 ± 2.48 mΩm

**Table 3:** Average resistivity of CNT film immersed in DI water and DI water/salts mixtures.

Test	Substance	Average Resistivity (Start) [µΩ]	Average Resistivity (24 h) [µΩ]	Resistivity Decrease
CNT film on glass in the mixture	NaCl	0.191 ± 0.029	0.168 ± 0.028	10–17%
CNT film	KCl	0.184 ± 0.044	0.160 ± 0.032	8–21%
CNT film on the glass in the air	-	0.184 ± 0.023	0.165 ± 0.016	5–15%
CNT film bridge in the mixture	KCl	0.142 ± 0.018	0.133 ± 0.014	−5–9%

## Data Availability

The datasets used and analyzed during the current study are available from the corresponding author upon request.

## Supplementary Materials

The following supporting information can be downloaded at: https://www.mdpi.com/article/10.3390/jfb15050113/s1, Figure S1: Defibrillation overload test circuit; Figure S2: Comparison of the average mass loss of hybrid CNT hydrogels produced by homogenizing CNTs in Acetone; Figure S3: ECG waveform of hybrid and reference electrode of V1 at the beginning of recording; Table S1: Resistance of paths screen printed with produced pastes in which the functional phase is CNTs homogenised with 40% and 60% power, depending on the homogenisation time; Table S2: Results of electrical test of hydrogels produced by mixing performed according to the ANSI/AAMI EC12:2000 (R2020) standard; Table S3: Comparison of the average mass loss after 6 h of 4 different batches of SignaGel and hybrid CNT hydrogels produced by homogenizing CNTs in IPA and CNT/SDS in DI water; Table S4: Results of impedance in frequency measurements of pure SignaGel hydrogel and hybrid SignaGel + 1% CNT (IPA); Table S5: Results of FFT analysis of ECG measurements at the beginning and after 72 h of recording; Table S6: Results of electrical tests of hybrid hydrogels produced by wrapping liquid hydrogel. Tests performed according to the ANSI/AAMI EC12:2000 (R2020) standard. The bolded values indicate that the results showed a statistically significant difference and the difference between the results exceeded 20%; Table S7: Results of electrical tests of hybrid hydrogels produced by wrapping solid structures performed according to the ANSI/AAMI EC12:2000 (R2020) standard.
