# Supplementary material for: Hydrogels and Carbon Nanotubes: Composite Electrode Materials for Long-Term Electrocardiography Monitoring
Source: J Funct Biomater. 2024 Apr 23;15(5):113. doi: 10.3390/jfb15050113 (PMC11122422; doi:10.3390/jfb15050113)
Supplement: Supplementary file 1 [file jfb-15-00113-s001.zip › jfb-2913582-supplementary.pdf]

Supplementary Material

Hydrogels and Carbon Nanotubes: Composite Electrode Materials for Long-Term Electrocardiography Monitoring

Leszek Kolodziej [1], Olga Iwasińska-Kowalska [1,*], Grzegorz Wróblewski [1], Tomasz Giżewski [2], Małgorzata Jakubowska [3,4] and Agnieszka Lekawa-Raus [4,*]

[1] Faculty of Mechatronics, Warsaw University of Technology, 02-525 Warsaw, Poland; leszek.kolodziej.dokt@pw.edu.pl (L.K.); grzegorz.wroblewski1@pw.edu.pl (G.W.)
[2] Faculty of Electrical Engineering and Computer Science, Lublin University of Technology, 20-618 Lublin, Poland; t.gizewski@pollub.pl
[3] Faculty of Mechanical and Industrial Engineering, Warsaw University of Technology, 02-524 Warsaw, Poland; malgorzata.jakubowska@pw.edu.pl
[4] Centre for Advanced Materials and Technologies, Warsaw University of Technology, 02-822 Warsaw, Poland
* Correspondence: olga.kowalska@pw.edu.pl (O.I.-K.); agnieszka.raus@pw.edu.pl (A.L.-R.)

According to ANSI/AAMI EC12:2000 (R2020) standard for disposable ECG electrodes following electrical test were performed:

- DCO Offset voltage measured after stabilization period (60-90s) of pairs of electrodes connected gel-to-gel shall be less than 100mV. Measurement input characteristics shall be 10nA or less bias current, 10Mohm or greater input impedance in the range 0 – 10Hz, input frequency response of at least 0.01 to 1000Hz.
- NOISE After the stabilization period, a pair of electrodes connected gel-to-gel shall not generate a voltage greater than 150 μVpp in the passband (first-order frequency response) of 0.15 to 100 Hz. Measurement shall be recorded after 1 minute from the start and shall record the maximum Vpp for the next 5 minutes.
- SDR This test measures the electrode's ability to reduce its acquired voltage, permitting the ECG trace to return after defibrillation, and shall be conducted, according to figure S1, as follows (once electrodes have been connected gel-to-gel and attached to the tester):

a) SW1 closed, SW2 and SW3 open – 200V charging cycle of the 10uf capacitor. Timing is between 10 – 20 sec.
b) SW1 and SW3 opened, SW2 closed – discharge to less than 2V in no longer than 2 sec.
c) SW1 and SW2 are opened, SW3 is closed – record DCO after 5 sec
d) DC0 is recorded every 10sec thereafter for the next 30 sec.
e) The electrode offset is recorded to the nearest 1 mV 5 sec after the closure of switch SW3 and every 10 sec thereafter for the next 30 sec. The overload and measurement are repeated three times.
f) For all electrode pairs tested, the 5-sec offset voltage after each of the four discharges of the capacitor shall not exceed 100 mV, and any difference in adjacent 10-sec values (after the initial 5-sec period) shall not exceed 10 mV.

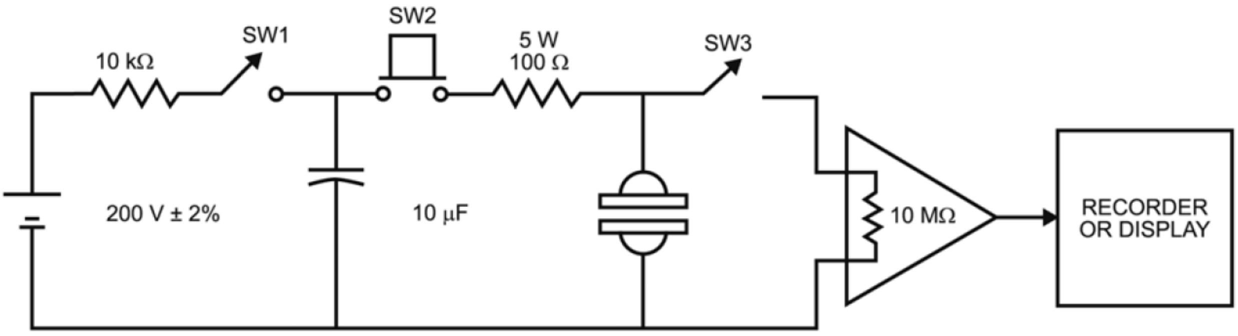

Figure S1. Defibrillation overload test circuit.

- BIAS The observed DC voltage offset change across a pair of electrodes connected gel-to-gel shall not exceed 100 mV when the electrode pair is subjected to a continuous 200 nA DC current over a period of not less than 8 hours. The current source shall use a voltage source of at least 2V. The differential voltage (measured voltage – initial voltage with no bias current) shall be recorded at least once per hour.

Table S1. Resistance of paths screen printed with produced pastes in which the functional phase is CNTs homogenised with 40% and 60% power, depending on the homogenisation time.

	Homogenisation power	Ultrasonic homogenisation time (min)					
		15	30	45	60	90	120
Average resistance of printed paths (kΩ)	40%	2650 ± 350	629 ± 172	279 ± 105	112 ± 18	91 ± 21	94 ± 24
	60%	46 ± 17	52 ± 17	148 ± 37	67 ± 17	61 ± 11	66 ± 18

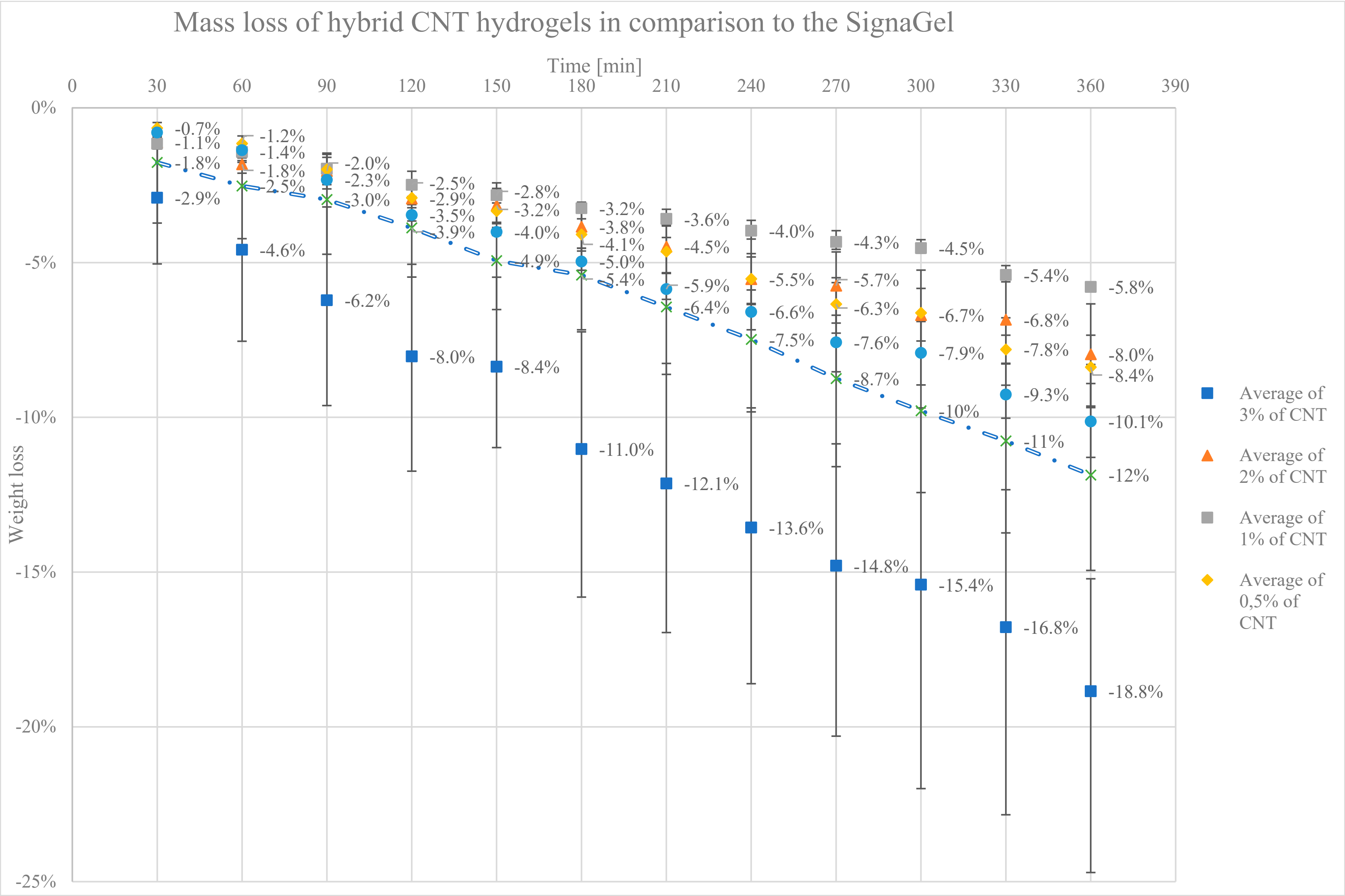

Figure S2. Comparison of the average mass loss of hybrid CNT hydrogels produced by homogenizing CNTs in Acetone

Journal of Functional Biomaterials

Table S2. Results of electrical test of hydrogels produced by mixing performed according to the ANSI/AAMI EC12:2000 (R2020) standard.

Test	SignaGel	SignaGel+ 1% CNT (IPA)	SignaGel+ 1% CNT (acetone)	SignaGel + 1% CNT/SDS (DI water)
1. DCO (mV)	0.1 ± 0.3	0.3 ± 0.4	-0.2 ± 0.6	0.1 ± 0.2
2. ACZ ǀ Impedance (Ohms)	**200 ± 40**	**60 ± 10**	**120 ± 40**	**130 ± 60**
3. SDR ǀ DCO (mV)/Slope (mV/Sec)	**5.0 ± 0.6**	**3.6 ± 0.6**	4.7 ± 1.3	4.7 ± 0.3
4. SDR ǀ DCO (mV)/Slope (mV/Sec)	5.2 ± 0.4	4.4 ± 0.5	5.1 ± 1.2	4.9 ± 0.5
5. SDR ǀ DCO (mV)/Slope (mV/Sec)	5.8 ± 0.3	5.0 ± 0.5	5.3 ± 0.9	5.6 ± 0.6
6. SDR ǀ DCO (mV)/Slope (mV/Sec)	6.3 ± 0.3	5.3 ± 0.6	5.6 ± 0.7	6.0 ± 0.7
7. ACZ ǀ Impedance (Ohms)	59 ± 19	46 ± 12	54 ± 12	53 ± 9
8. NOISE ǀ Amplitude (µVpp)	40.1 ± 1.8	41.3 ± 5.1	51 ± 10	39.3 ± 10
9. BIAS (mV)	1.1 ± 0.5	1.4 ± 1.3	0.3 ± 0.3	0.6 ± 0.3

Table S3. Comparison of the average mass loss after 6 hours of 4 different batches of SignaGel and hybrid CNT hydrogels produced by homogenizing CNTs in IPA and CNT/SDS in DI water.

Product	Average mass loss after 6 hours
SignaGel batch 1	-6.3 % ± 1.2 %
SignaGel batch 2	-7.6 % ± 4.6 %
SignaGel batch 3	-4.2 % ± 1.4 %
SignaGel batch 4	-4.3 % ± 0.7 %
Hybrid hydrogel CNT (IPA)	-4.5 % ± 1.5 %
Hybrid hydrogel CNT/SDS (DI water)	-5.6 % ± 1.3 %

Table S4. Results of impedance in frequency measurements of pure SignaGel hydrogel and hybrid SignaGel + 1% CNT (IPA).

Frequency [Hz]	Impedance [Ω]					
	Pure SignaGel			Hybrid hydrogel		
**0,52**	1535,9	1032,4	755,0	394,5	351,2	373,2
**0,67**	1343,6	930,3	729,6	373,3	337,4	371,9
**0,72**	1281,0	885,0	722,5	363,2	329,6	373,9
**0,78**	1223,1	839,3	710,6	360,4	321,5	365,6
**1,17**	966,1	679,9	636,4	320,5	285,7	327,1
**1,27**	919,9	644,8	620,1	313,3	282,5	317,4
**1,38**	876,8	611,6	603,4	300,5	273,7	313,8
**1,90**	724,9	522,4	535,5	266,3	242,1	278,6
**2,24**	660,0	470,9	503,3	244,3	224,9	258,9
**2,43**	629,6	446,5	488,0	232,3	219,0	252,5
**3,09**	548,0	403,3	444,9	211,1	194,6	224,6
**3,35**	524,2	383,0	431,3	200,2	188,4	217,8
**3,94**	480,3	346,9	405,6	180,6	173,4	200,7
**5,02**	422,1	314,5	371,9	162,3	153,3	178,1
**5,45**	404,9	299,6	361,8	153,6	146,7	170,7
**5,91**	388,6	285,6	351,9	145,3	140,5	163,6
**8,16**	332,2	248,8	317,3	123,4	118,0	138,6
**8,85**	319,8	238,0	309,7	116,8	112,8	132,8
**9,59**	308,2	227,7	302,6	110,7	107,9	127,6
**10,40**	297,2	218,2	296,0	104,9	103,1	122,4
**12,23**	276,9	209,3	283,6	99,5	94,3	112,5

**13,26**	267,6	200,9	278,0	94,4	90,1	107,9
**14,37**	258,7	193,0	272,7	89,7	86,1	103,6
**15,58**	250,3	185,7	267,7	85,2	82,3	99,4
**18,32**	234,9	178,9	258,7	77,2	75,2	91,6
**21,54**	221,1	166,5	250,6	73,6	68,8	84,6
**23,35**	214,7	160,9	247,0	70,2	65,7	81,4
**27,45**	203,1	155,7	240,5	64,0	60,1	75,3
**32,27**	192,6	146,5	234,7	61,3	55,0	69,9
**34,99**	187,7	142,3	232,1	58,7	52,7	67,4
**37,93**	183,1	138,4	229,6	56,3	50,4	65,0
**48,35**	170,9	131,5	223,2	51,9	44,3	58,4
**52,42**	167,2	128,4	221,3	50,0	42,5	56,4
**56,84**	163,7	125,5	219,5	48,2	40,7	54,6
**61,62**	160,5	122,8	217,8	46,5	39,1	52,8
**66,81**	157,4	120,3	216,3	44,9	37,5	51,1
**78,54**	151,8	118,0	213,5	43,4	34,6	48,0
**85,16**	149,2	115,8	212,2	42,0	33,2	46,5
**92,33**	146,8	113,8	211,0	40,7	32,0	45,1
**100,11**	144,6	112,0	209,9	39,5	30,7	43,8
**127,60**	138,7	108,7	206,9	37,4	27,5	40,2
**138,35**	137,0	107,2	206,0	36,4	26,5	39,2
**150,00**	135,4	105,9	205,2	35,5	25,6	38,1

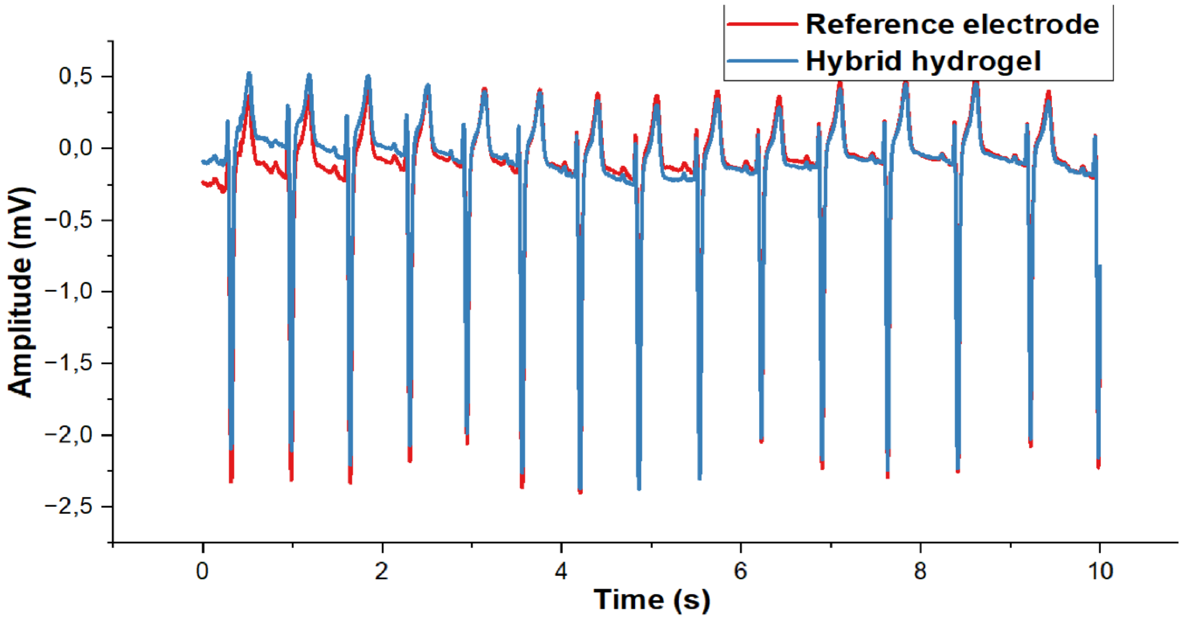

Figure S3. ECG waveform of hybrid and reference electrode of V1 at the beginning of recording.

Table S5. Results of FFT analysis of ECG measurements at the beginning and after 72 hours of recording.

Time, electrode	Base frequency [Hz]	1 harmonic	2 harmonic	3 harmonic	4 harminic	5 harmonic	6 harmonic	7 harmonic
0h C1 Hybrid hydrogel	1,89981	1	0,377629	0,335099	0,234846	0,908637	0,25844	0,412207
0h C1 Reference electrode	1,89981	1	0,399542	0,413731	0,488434	0,372804	0,193389	0,121098
0h C2 Hybrid hydrogel	1,89981	1	0,339905	0,359307	0,321064	0,220295	0,124927	0,096792
0h C2 Reference electrode	1,89981	1	0,307872	0,338864	0,277767	0,179585	0,107783	0,085444
0h C3 Hybrid hydrogel	1,9998	1	0,451025	0,112906	0,130162	0,032442	0,048464	0,176349
0h C3 Reference electrode	1,9998	1	0,4269	0,107266	0,126338	0,037489	0,036328	0,162214

72h C1 Hybrid hydrogel	1,79982	1	0,449363	1,784239	5,270975	0,471117	0,398629	1,893797
72h C1 Reference electrode	1,79982	1	0,472818	1,866408	5,463982	0,471843	0,407351	1,933169
72h C2 Hybrid hydrogel	1,79982	1	0,241944	1,543007	4,176674	0,351828	0,231648	1,259385
72h C2 Reference electrode	1,79982	1	0,151493	1,267021	3,217242	0,280793	0,134816	0,801349
72h C3 Hybrid hydrogel	1,9998	1	0,484335	0,286174	2,836424	0,411855	1,14938	0,879259
72h C3 Reference electrode	1,9998	1	0,485729	0,289353	2,763781	0,338433	1,041108	0,830938

Table S6. Results of electrical tests of hybrid hydrogels produced by wrapping liquid hydrogel. Tests performed according to the ANSI/AAMI EC12:2000 (R2020) standard. The bolded values indicate that the results showed a statistically significant difference and the difference between the results exceeded 20%.

Test	SignaGel	SignaGel wrapped in CNT film
1. DCO (mV)	0.1 ± 0.3	0.1 ± 0.1
2. ACZ ǀ Impedance (Ohms)	**200 ± 40**	**14 ± 52**
3. SDR ǀ DCO (mV)/Slope (mV/Sec)	**5.0 ± 0.6**	**0.2 ± 0.6**
4. SDR ǀ DCO (mV)/Slope (mV/Sec)	**5.2 ± 0.5**	**0.2 ± 0.6**
5. SDR ǀ DCO (mV)/Slope (mV/Sec)	**5.8 ± 0.4**	**0.1 ± 1.3**
6. SDR ǀ DCO (mV)/Slope (mV/Sec)	**6.3 ± 0.3**	**0.1 ± 0.1**
7. ACZ ǀ Impedance (Ohms)	**58 ± 20**	**16 ± 53**
8. NOISE ǀ Amplitude (μVpp)	40.1 ± 1.8	39. 4 ± 9
9. BIAS (mV)	**1.1 ± 0.5**	**0.1 ± 0.1**

Table S7. Results of electrical tests of hybrid hydrogels produced by wrapping solid structures performed according to the ANSI/AAMI EC12:2000 (R2020) standard.

Test	KM30B	KM30B + CNT film	Foam + CNT film
1. DCO (mV)	**1.3 ± 6**	0.0± 0.2	0.1 ± 0.3
2. ACZ ǀ Impedance (Ohms)	**295 ± 190**	**220 ± 140**	**190 ± 140**
3. SDR ǀ DCO (mV)/Slope (mV/Sec)	**8.4 ± 7.8**	**0.1 ± 0.3**	**0.2 ± 0.3**
4. SDR ǀ DCO (mV)/Slope (mV/Sec)	**8.8 ± 7.9**	**-0.1 ± 0.2**	**0.1 ± 0.2**
5. SDR ǀ DCO (mV)/Slope (mV/Sec)	**9.2 ± 7.9**	**0.0 ± 0.2**	**0.1 ± 0.2**
6. SDR ǀ DCO (mV)/Slope (mV/Sec)	**9.5 ± 7.9**	**0.1 ± 0.2**	**0.1 ± 0.2**
7. ACZ ǀ Impedance (Ohms)	**216 ± 116**	**80 ± 56**	**22 ± 56**
8. NOISE ǀ Amplitude (μVpp)	**105 ± 46**	**28 ± 56**	**38 ± 56**
9. BIAS (mV)	**8.2 ± 0.3**	**0.1 ± 0.4**	**0.2 ± 0.4**
